# Development of a robust DNA damage model including persistent telomere-associated damage with application to secondary cancer risk assessment

**DOI:** 10.1038/srep13540

**Published:** 2015-09-11

**Authors:** Soheil Rastgou Talemi, Gabriel Kollarovic, Anastasiya Lapytsko, Jörg Schaber

**Affiliations:** 1 Institute for Experimental Internal Medicine, Medical Faculty, Otto von Guericke University, Magdeburg, Germany; 2 Cancer Research Institute, Slovak Academy of Sciences, Bratislava, Slovakia

## Abstract

Mathematical modelling has been instrumental to understand kinetics of radiation-induced DNA damage repair and associated secondary cancer risk. The widely accepted two-lesion kinetic (TLK) model assumes two kinds of double strand breaks, simple and complex ones, with different repair rates. Recently, persistent DNA damage associated with telomeres was reported as a new kind of DNA damage. We therefore extended existing versions of the TLK model by new categories of DNA damage and re-evaluated those models using extensive data. We subjected different versions of the TLK model to a rigorous model discrimination approach. This enabled us to robustly select a best approximating parsimonious model that can both recapitulate and predict transient and persistent DNA damage after ionizing radiation. Models and data argue for i) nonlinear dose-damage relationships, and ii) negligible saturation of repair kinetics even for high doses. Additionally, we show that simulated radiation-induced persistent telomere-associated DNA damage foci (TAF) can be used to predict excess relative risk (ERR) of developing secondary leukemia after fractionated radiotherapy. We suggest that TAF may serve as an additional measure to predict cancer risk after radiotherapy using high dose rates. This may improve predicting risk-dose dependency of ionizing radiation especially for long-term therapies.

Mathematical models of DNA damage dynamics have been instrumental to understand mechanisms and kinetics of ionizing radiation (IR)-induced DNA damage repair for over 60 years^1^,^2^,^3^,^4^. Such rather phenomenological models for dose-response relationship form the base for IR-associated carcinogenesis risk assessment, specifically after radiotherapy^5^,^6^,^7^,^8^, and also for IR-induced cancer on long-term space flights^9^. To describe the dynamics of the most severe type of DNA damage, i.e. double strand breaks (DSBs), the two-lesion kinetic (TLK) model is widely accepted^1^,^10^. This model proposes two kinds of DSBs, simple and complex ones, which are repaired with different rates. It has been argued that some simple DSBs contain additional elementary damage sites (base damage, strand breaks, base deletion, etc.) within the same section of DNA, which renders them more complex, and therefore longer, to repair^1^. Both types of DSBs are principally repaired following a first-order kinetic. In one study half-lives of etoposide-induced γH2AX foci have been determined to be ≈2 h for around 90% of foci and ≈12 h for 10% of foci, clearly indicating two different kinds of DNA damage^11^.

However, it has been noted that first-order kinetics are not sufficient to explain observed DNA damage dynamics. Therefore, second-order mechanisms have been postulated^2^,^4^,^12^, also referred to as linear-quadratic (LQ) formalism^2^,^4^,^12^. This mechanism is mainly attributed to repair events in which break ends not associated to the same DSB are involved. However, different repair pathways with different efficiency and preference to cell cycle stage may also be involved in this effect^11^,^13^,^14^. The former, so-called binary misrepair events, may lead to lethal DNA damage, because they are linked to various classes of intra- or interchromosomal (complete or incomplete exchange-type) aberrations, e.g. dicentrics, acentric rings, and translocation aberrations^1^,^2^.

The classical TLK model including second order mechanisms was extended by considering a possible limiting effect of repair enzymes and their complexes^10^,^15^. Especially, when many DSBs occur at once due to IR, the amount of repair enzymes and their complexes might not suffice to repair all DSBs in parallel. Therefore, the rate at which DSBs are repaired might saturate at elevated levels of DNA damage.

Recently, it was shown that apart from simple and complex DNA damage, persistent DNA damage is a third kind of IR-induced DNA damage^16^, evidenced by persistent γH2AX foci for up to four months^17^,^18^. These persistent DSBs have been shown to be associated with telomeres and, thus, will in the following be referred to as telomere-associated foci (TAF)^17^,^18^. There is strong evidence that telomeres-associated DSB in circular DNA cannot be repaired, a mechanism that usually prevents chromosome fusions^16^,^17^,^18^,^19^,^20^. Consequently, the number of persistent telomere-associated DNA damage increases with increasing radiation dose^17^,^18^. Such persistent DNA damage is important for radiotherapy applications where accumulated doses are high. To incorporate these recent findings into existing models of DNA damage dynamics, we extended the TLK model by additional categories of DSBs, namely telomere-associated DNA damage foci (*T*_AF_) and basal background damage (*B*) and validated the extended DNA damage model with measured data (note that TAF is an acronym, whereas *T*_AF_ refers to a model variable (Fig. 1)). Basal background damage (*B*) was newly introduced based on the observation that there is always a certain level of γH2AX foci irrespective from IR-treatment. These foci may be due to background IR coming from natural sources or cell-intrinsic DNA damaging factors, like reactive oxygen species. In addition, we compared different versions of the TLK model also including the suggested extension by repair proteins leading to saturable repair kinetics^10^. Apart from the simplified process of repair enzyme binding, all other biochemical details are lumped into the model parameters (Fig. 1). Therefore, the parameters cannot directly be linked to molecular entities or processes.

For validation we fitted 20 candidate models to measured time series of DSBs and *TAF*. Here, we use the accepted measure of γH2AX foci number as a readout for DSBs^21^. We wanted to identify a parsimonious model, which can best explain observed dynamics of DSBs and that is as simple as possible, yet as complex as necessary. To this end, we subjected all models to a rigorous model discrimination approach in which we ranked all models according to the Akaike Information Criterion corrected for small sample size (AICc) that measures parsimonious data representation.

We could robustly select a best approximating parsimonious model that can both recapitulate and predict transient and persistent telomere-associated DNA damage after ionizing radiation. The data support a model with i) nonlinear dose-damage relationships, and ii) negligible saturation of repair kinetics even for high doses. Additionally, we show that simulated radiation-induced persistent telomere-associated DNA damage foci (TAF) can be used to predict excess relative risk (ERR) of secondary leukemia after fractionated radiotherapy.

## Results

### Model candidates

**Figure 1:**
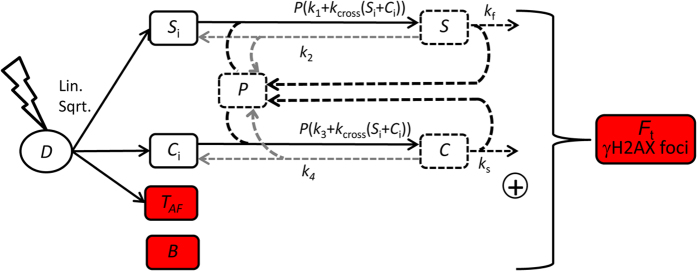
Conceptual candidate models. Ionising radiation dose (*D*) induces initial simple and complex DSBs (*S*_i_ and *C*_i_, respectively) as well as persistent telomere-associated DNA damage foci (*T*_*AF*_), in addition to the basal background damage (*B*). The initial foci can bind repair proteins (*P*) forming complexes (*S* and *C*, respectively), which are subsequently repaired. For simplicity only first order reactions are depicted as arrows. Second-order reactions are indicated in the rate law formulas for the binding reactions. The sum of all damage components (*F*_t_ = *B* + *T*_*AF*_ + *C* + *C*_i_ + *S* + *S*_i_) constitutes the measured γH2AX foci number as readout for DSBs. Dashed components and reactions indicate alternative model formulations. Black components are included in the best selected model, whereas grey components were not included. Colored components are the new components we added to the two lesion kinetic model suggested by Ma *et al.* (2005).

Starting point for the candidate models is the two-lesion kinetic (TLK) model including saturable kinetics by repair enzyme binding^10^. Accounting for new insights about telomere-associated DNA damage repair outlined above, we extended this model with additional DSB categories, namely telomere-associated damage foci (*T*_AF_) and basal background damage (*B*) (Fig. 1). This model constituted the most complex one with maximally 13 parameters and six variables. All other tested models are simpler versions of this model.

Ionizing radiation dose (*D*) initially induces three types of DSBs: simple DSB (*S*_i_), complex DSB (*C*_i_), and telomere-associated DNA damage foci (*T*_AF_) in addition to the basal background damage (*B*) (Fig. 1). The induced simple and complex DSBs can be repaired by a) first-order kinetics (*k*_1_ and *k*_3_), and, optionally, by b) second-order kinetics (*k*_cross_(*S*_i_ + *C*_i_)). The initial first and optional second order kinetics can be reversibly modified by repair protein complexes (*P*) of fixed amount, leading to simple and complex protein-bound DSBs, *S* and *C*, respectively. These protein-bound DSBs are subsequently repaired with first-order kinetics (*k*_f_ and *k*_s_) (Fig. 1). In the process of model fitting we noticed that the backward reactions of repair-protein binding (*k*_2_ and *k*_4_) were poorly identifiable (Figure S3) and were, therefore, treated as optional as well. Initially, we use the same fixed amount of repair proteins as in a published model^10^. Alternatively, we allowed this amount to be a free parameter in the optimization procedure. We assume that persistent telomere-associated DNA damage foci (*T*_AF_) cannot be repaired within the considered time.

We had to define a relation between ionizing radiation (*D*) and initially induced DSBs. Generally, this relation is assumed to be linear^1^,^2^,^10^ and there are several measurements that support this notion for MRC5 normal human fibroblast that we used here^22^,^23^. However, for *T*_AF_ we found that a square-root relation fitted better to the available data (Figure S1). Therefore, we alternatively included square-root relations for all three types of IR-induced DSBs.

In total, we tested 20 candidate models. The candidate models were implemented as system of ordinary differential equations (Supplementary Materials, Model Formulation).

### Data

**Figure 2:**
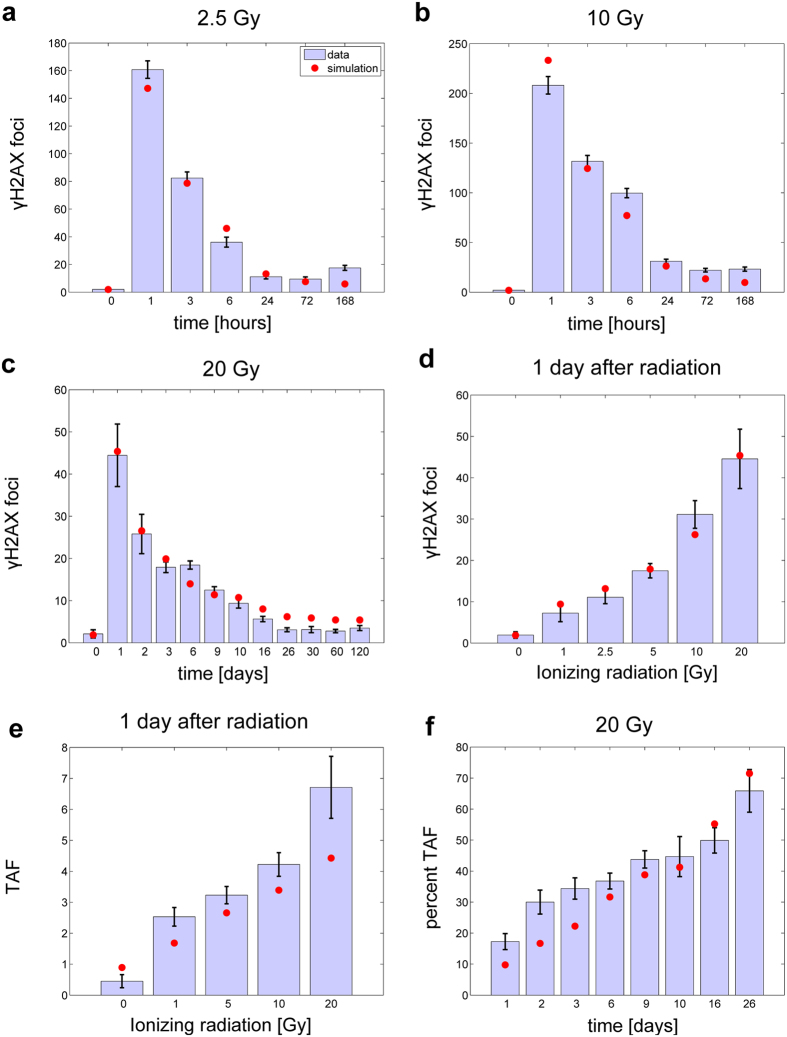
Data and simulations. (**a**) Measured mean ± standard error of the mean (SEM) γH2AX foci per cell (bars) and simulations of the number of DSBs (*F*_t_) (filled circles) of the best approximating model (Nr. 10) for 2.5 Gy over time. (**b**) Measured mean ± SEM γH2AX foci per cell (bars) and simulations of the number of DSBs (*F*_t_) (filled circles) of the best approximating model (Nr. 10) for 10 Gy over time. (**c**) Measured mean ± SEM γH2AX foci per cell (bars) and simulations of the number of DSBs (*F*_t_) (filled circles) of the best approximating model (Nr. 10) for 20 Gy over time. (**d**) Measured mean ± SEM γH2AX foci per cell (bars) and simulations of the number of DSBs (*F*_t_) (filled circles) of the best approximating model (Nr. 10) one day after radiation for different doses. The 2.5Gy data point was obtained from a linear interpolation between 1 and 5Gy. (**e**) Measured mean ± SEM TAF per cell (bars) and simulations of TAF (*T*_AF_) (filled circles) of the best approximating model (Nr. 10) one day after radiation for different doses. (**f**) Measured mean ± SEM TAF per cell (bars) and predictions of percent TAF (*T*_AF_) (filled circles) of the best approximating model (Nr. 10) for 20 Gy over time. Data in Fig. a,b were measured for this project. Data in Fig. c were combined from^17^,^18^, data in Fig. d–f were digitized from^18^.

For model parameterization, we compiled data for IR-induced γH2AX foci, an accepted measure for DSBs, for MRC5 primary human fibroblasts from three different sources. First, we measured γH2AX foci time series ourselves, for 2.5 and 10 Gy (Fig. 2A,B, Method Section). Second, we combined digitized γH2AX foci and TAF time series for MRC5 cells from Hewitt *et al.* (2012) and Fumagalli *et al.* (2012) to derive an extended consensus γH2AX foci time series for 20 Gy (Fig. 2C). All other data were taken from Hewitt *et al.* (2012) (Fig. 2D–F, S2A). Our foci quantification procedure and data scaling is described in the Methods Section.

### Model selection and cross-validation

We fitted the models to the available data (Fig. 2A–E, S2) and ranked them according to the Akaike Information Criterion corrected for small sample size (AICc) (see Materials and Methods section) (Table 1)^24^.

The most probable candidate models, according to the AICc, were those models that employed both second-order kinetics as well as repair protein binding. In addition, almost all models that assumed a square-root relation between absorbed radiation dose *D* [Gy] and initial DSBs were ranked before those that assumed a linear relation. The best approximating model Nr. 10 had no repair protein-DSB complex dissociation reaction and used a previously suggested amount of repair proteins. However, the same model only with the amount of repair proteins as a free parameter (Model Nr. 18) was ranked second and the according fitted value was close to the suggested one. The list of the fitted parameters can be found in the online Supplementary Table S1. The parameterized model Nr. 10 with corresponding data can also be found in the online Supplementary Material in COPASI- and SBML-format as well as in the BioModels database^25^ (access number: MODEL1412200000). The best approximating model Nr. 10 could also well predict the increasing percentage of TAF over time (Fig. 2F).

**Figure 3:**
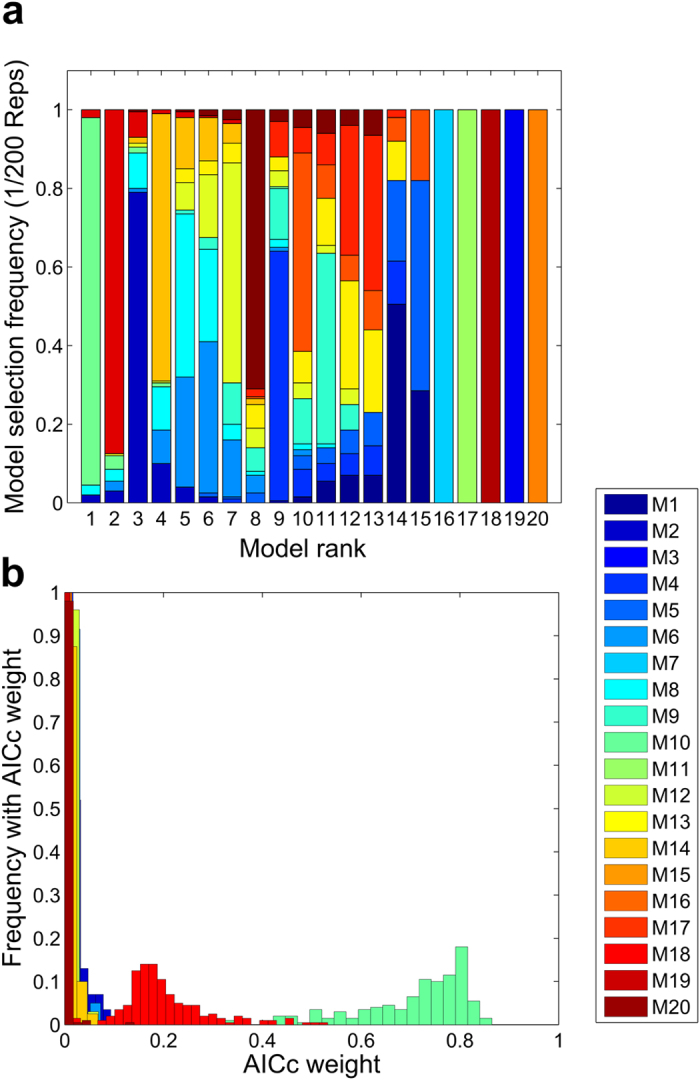
200 independent model selection trials were conducted using resampled data. Data resampling was done assuming that DNA damage foci are Poisson distributed. This analysis strongly favours model (Nr. 10) as the most probable-parsimonious model in the ensemble. (**a**) Frequency of achieving a specific rank for each model, after 200 independent model selection trials, is depicted versus the corresponding rank number. Model Nr. 10 was ranked first in more than 90% of model selection trials, model Nr. 18 dominated rank 2 and model Nr. 2 rank 3. (**b**) Frequency of achieving a specific AICc weight out of 200 independent model selection trials, for each model, is depicted versus AICc weights. Model Nr. 10 AICc weight is mainly distributed over large values, 0.7–0.8, whereas its strongest rival AICc weight (Model Nr. 18) is distributed between 0.1–0.3. Other model’s AICc weight is mainly distributed between 0–0.1.

AICc model ranking is known to be sensitive to uncertainty in the data^26^. Thus, to check plausibility and robustness of the model selection, we conducted a Monte Carlo analysis. We generated 200 random data sets from original data (Fig. 2A–E) assuming that radiation induced DSB foci are distributed according to Poisson distribution^2^,^23^,^27^ using experimental data points as respective averages. Subsequently, we fitted the models to each data set and ranked them using the AICc. These 200 independent model selection trials provided statistics for the assessment of model selection plausibility and robustness. We analyzed model selection frequency, calculated as the number of times a model achieved a specific rank out of 200 trials, and AICc weight distribution (Materials and Methods, model discrimination). The model selection frequency and AICc weight distribution also suggested model Nr. 10 as the by far most probable parsimonious model in the ensemble (Fig. 3).

We also assessed the predictive power of the models using a simple cross validation approach. Here, we calculated the average prediction error of each model for five different cross-validation runs, in which we used one of the data sets in Fig. 2A–F (excluding TAF data) for prediction and the remaining for calibration, respectively (Materials and Methods, cross-validation). Model Nr. 18 and Nr. 10 had the best predictive power, respectively (Table 2). Given the fact that Model 18 is structurally the same as Model Nr 10 only using the amount of repair proteins as a free parameter and the fact Model Nr. 10 was robustly selected as best approximating model above, we selected Model Nr. 10 as the final best approximating model for reasons of parsimony.

### Parameter identifiability

**Figure 4:**
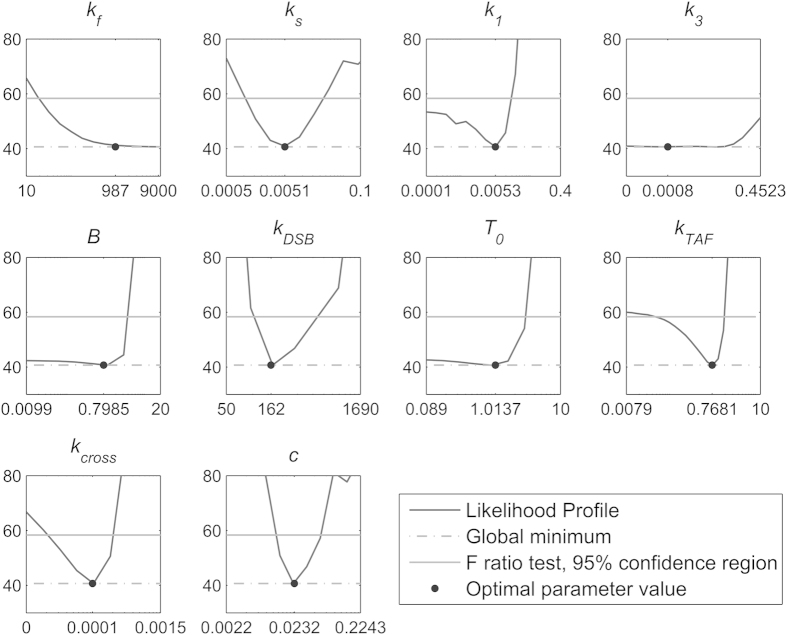
Likelihood profile-based parameter identifiability analysis for model Nr. 10. The sum of squared residuals (SSR) after parameter estimation is plotted versus the scanned parameter values. 95% confidence region is calculated by F ratio test (grey solid line). The minimum objective value reached is shown at bottom (grey dashed line) and the corresponding estimated parameter value is shown by a bold dot.

To explore whether the parameter estimation led to identifiable parameter values, we conducted a likelihood profile-based identifiability analysis^28^ using COPASI^29^ for the best ranked model Nr. 10. Model parameters with a well-defined likelihood-based confidence interval, indicated by a likelihood profile ultra-passing the 95% confidence threshold (grey solid lines in Figures S3, S4), can be uniquely defined within that interval and are called practically identifiable. Parameters whose likelihood profiles do not ultra-pass the 95% confidence threshold from both sides, but are not completely flat, are called structurally identifiable, because having appropriate data they will eventually become practically identifiable. Structurally non-identifiable parameters cannot be uniquely defined independent from data^30^. Parameter identifiability analysis showed that five out of ten (50%) parameters were practically identifiable and all parameters were practically identifiable at least from one side (Fig. 4). The percentage of identifiable parameters of the two best ranked models with maximum and minimum number of parameters, model Nr. 14 and model Nr. 8, was 30 and 70%, respectively (Figures S3, S4). In addition, all of the models, even the most complex model in the ensemble, showed structural identifiability.

### Robustness analysis

**Figure 5:**
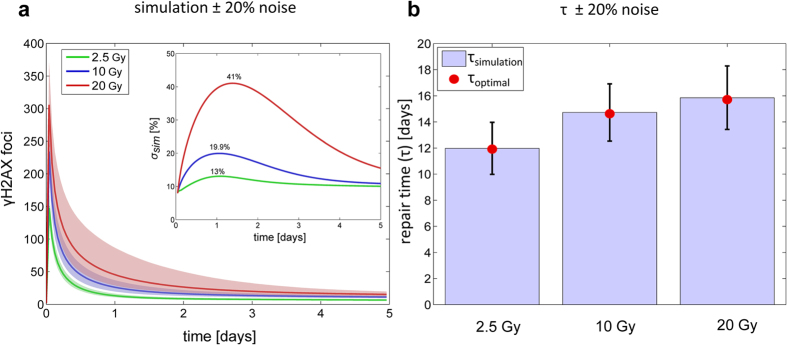
Robustness analysis of the best approximating model Nr. 10 with respect to perturbations in the estimated parameters. Model parameters were perturbed ± 20%. (**a**) Main panel: Total number of DSBs is simulated for 2.5, 10 and 20 Gy versus time (solid line). The shading region indicates 5–95% quantiles of respective model simulations. Inset: the standard deviation of the simulations from optimal solution, *σ*_*sim*_, is plotted versus time. The maximum *σ*_*sim*_s were 13, 19.9 and 41% for 2.5, 10 and 20 Gy IR, respectively. (**b**) Estimated repair times τ [days] for different IR doses, 2.5, 10 and 20 Gy, respectively. Bars showing average τ values from 1000 simulations, τ_simulation_, with standard deviations as error bars. The τ value of the respective optimal solution, τ_optimal_, is shown by filled circles.

We studied the robustness of the best approximating model Nr. 10 with respect to noise in its parameters (Materials and Methods, robustness analysis). We conducted 1000 simulations assuming a parameter uncertainty of ±20% around their fitted values using Latin hypercube sampling. The uncertainty of simulated γH2AX values increases with radiation dose, especially within the first days after irradiation (Fig. 5a-main panel). We quantified the uncertainty of the simulated H2AX values by the standard deviation of the simulations relative to the optimal solution.

This relative standard deviation *σ*_*sim*_(*t*) varied between 10% and maximally 13, 19.9 and 41% for 2.5, 10 and 20 Gy IR, respectively (Fig. 5a-inset). The repair time *τ*, defined as the time when the simulated γH2AX foci reached 150% of their respective final value, showed a standard deviation of about ±2 days from its optimal value for all IR doses. Thus, the selected model Nr. 10 proved to be robust with respect to noise in its parameters.

### Role of repair protein binding

The analysis of the best approximating model revealed that there was no saturation of the repair rate because of repair protein depletion even after excessive DNA damage (Figure S5A). Two parameters turned out to be sensitive with respect to repair protein depletion; *c*, the percentage of initial complex DSBs, and *k*_f_, the repair rate of simple repair-protein bound DSBs (Figure S5B-C). When the repair rate is saturated because of depleted repair proteins, the decrease of γH2AX foci with time becomes linear (Figure S5B-C). This is evidenced neither by models nor the data (Fig. 2A–C). Thus, we find no support for the notion that the repair rate of DSBs becomes saturated or repair proteins are depleted, at least under the conditions and within the time frames tested here. This poses the question, why the models including repair proteins were clearly favoured by the model selection procedure despite the fact that saturation plays no role. Apparently, the two additional parameters provide a sufficiently increased flexibility to the model such that especially the amount of γH2AX foci 1 day after IR could be better fitted by the model with repair-protein binding (Nr. 10) compared to the best ranked models without repair-protein binding (Nr. 6 and 8) (Figure S5D).

### Predicting cancer risk after radiotherapy

**Figure 6:**
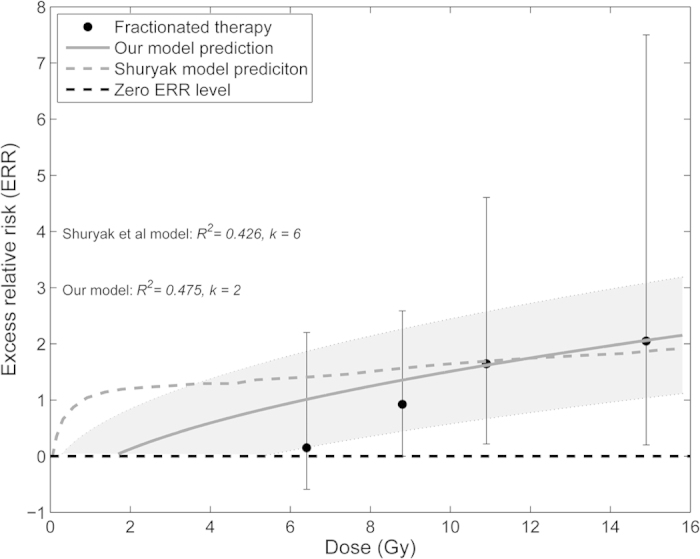
The excess relative rate (ERR) of developing secondary leukaemia in patients with cancer of uterine corpus receiving fractionated radiation therapy. ERR is plotted versus the cumulative radiation dose (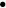). Our model prediction (solid line) was calculated with the formula ERR = T_AF+_ − *B* using fitted parameters from the best approximating model Nr. 10 (Table S1). Although our model has fewer number of free parameters (k = 2 vs 6) it predicts the ERR better than Shuryak *et al.* model (*R*^*2*^ = 0.475 vs 0.426). Shading represents 95% confidence interval of the simulated mean with respect to estimated parameters *B* and *k*_*TAF*_, using our model (model Nr. 10). Data and Shuryak *et al.* model prediction were digitized from published results^8^.

Persistent DNA damage has been related to a cellular senescence phenotype^16^,^17^,^18^. Senescent cells exhibit a secretory phenotype^31^,^32^, which in turn has been related to chronic inflammation and cancer^33^,^34^. Moreover, persistent telomeric DNA damage has also directly been linked to cancer^35^. We therefore wondered whether our predicted persistent telomeric DNA damage foci might be related to increased cancer risk after radiotherapy. To test this idea, we analysed data for secondary leukemia risk after radiotherapy of uterine corpus cancer measured as excess relative risk (ERR)^36^. ERR estimates the risk of leukemia after radiotherapy compared to a matched control. This estimate is based on a cohort of 110 000 women with invasive cancer of the uterine corpus who survived at least 1 year following their initial cancer that was assembled from nine population-based cancer registries (see Curtis *et al.* (1994) for a detailed description of the data). This data was used before to test sophisticated models for dose-risk relationship^8^ (Fig. 6). We expected that there might be a threshold of radiation induced telomeric foci beyond which an elevated cancer risk would become measurable. Interestingly, we found that measured excess relative risk (ERR) of secondary leukemia after fractionated radiotherapy could be well predicted by simulated radiation-induced TAF corrected for background DNA damage *B*:


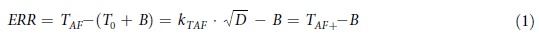



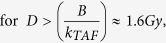


where *T*_AF+_ are additional radiation-induced telomeric foci excluding background TAF (*T*_0_). Persistent DNA damage of surviving cells after radiotherapy can be well predicted by our simple model for TAF. Therefore, our model may be a valuable and simple measure for increased cancer risk after radiotherapy.

## Discussion

Mathematical models of ionizing radiation (IR)-induced double strand breaks (DSBs) have been used to understand the mechanism of DNA damage/repair as well as estimation of radiation-associated secondary cancer risks^1^,^2^,^3^,^4^,^5^,^6^,^7^,^8^. Here, we studied DSBs dynamic in MRC5 primary human fibroblast cells under different ionizing radiation doses. In this cell type, cell death even for doses as high as 20 Gy is reported to be negligible^37^, which we could confirm as well (Figure S6). MRC5 and other primary fibroblast rather go to senescence instead to apoptosis after being treated with IR^16^,^17^,^18^. Thus, it is possible to track DNA damage dynamics for up to four months (Figure 2C).

We developed and parameterized an ensemble of models for IR-induced DNA double strand break (DSB) repair kinetics based on the existing and well-established two-lesion kinetics (TLK) model. For the first time, these models also included terms for constant background damage and IR-induced persistent telomere-associated damage foci (TAF). Our best approximating model was able to well recapitulate available time series of both total and telomere-associated DSBs under various radiation regimes (Fig. 2A–E). Moreover, it also well predicted the percentage of TAF over time, which was measured independently and was not used to parameterize the model (Fig. 1F). The practical identifiability of the model parameter *c*, the percentage of the complex DSBs, and our model discrimination approach clearly argues for the existence of a) two distinct classes of DSBs with different repair rates, and, b) a second-order repair mechanism. This supports the classical two-lesion-kinetic (TLK) model, where the second-order mechanism has been mainly attributed to so-called binary misrepair events^2^,^4^,^12^, but could also be related to different repair pathways being active at different stages of the cell cycle^13^,^14^, or both. Our model suggests that around 2% of initially induced DSBs are complex. (Table S1). Around 10% was estimated by previous reports^11^. As our estimate was practically identifiable (Fig. 4) and the 95% confidence interval ranges from 1.6% to 2.8% we conclude that earlier reports might overestimate the amount of complex DSBs.

It should be noted that our extended TLK model is designed to explain average DNA repair kinetics in a phenomenological manner and, therefore, lacks mechanistic details, which are difficult to parameterize. It also neglects feedback mechanisms that have been shown to play a role in radiation induced DNA damage. Is has been shown, that irradiated MRC5 cells produce reactive oxygen species (ROS) contributing significantly to short-lived DNA damage but non-significantly to long-lived (>15 h) DNA damage^38^. However, the long-term effect (>2d) it is unclear. In addition, even though telomeric DNA damage has been called non-repairable^17^, there is still the possibility that some foci are eventually repaired with a very slow kinetic. Thus, elevated average persistent DNA damage might also be a net effect of repaired and freshly induced TAF, e.g. by feedback-mediated ROS. These processes are lumped together in our simple phenomenological model, which supports the notion that, once induced, TAF stay constant on the population average for the time considered here.

We also included the possibility of repair-protein binding forming protein-DSB complexes, which can be repaired by a first order process. The models including this simple mechanistic detail were clearly favoured by the model selection procedure. Repair-protein binding was initially proposed to allow for saturating effects brought about by condition in which the number of DSBs exceeds the number of repair-proteins and their complexes^10^,^15^. Interestingly, our models were parameterized in a way such that under the studied conditions saturation of the repair process because of repair-protein depletion did not occur. This was also not supported by the data in which a linear decrease of γH2AX foci with time indicating a saturated process was not substantiated. The reason why these models were selected despite missing saturation effect was the fact that the two additional parameters for the repair of the DSB-protein complexes provided an increased flexibility such that the data could be better explained. This applies especially to γH2AX foci 24 h after radiation (Figure S2D) that contributed substantially to the overall sum of squared residuals. We cannot exclude the possibility that DSB repair might become saturated shortly after high IR regimes. Our data starts only one hour after radiation. Therefore, at earlier time points saturation effects might be observable. However, we were unable to meaningfully measure earlier time points, because with the available technology the measured γH2AX foci would saturate at earlier time points, especially for 10 Gy (Figure S7, S8). The lack of data for time points earlier than 1 h is also the reason why the repair rate for simple protein-DSB complexes (*k*_f_) was not practically identifiable. However, all parameters of the best approximating model Nr. 10 and of the best ranked models with maximum and minimum number of parameters, model Nr. 14 and model Nr. 8, were identifiable at least from one side. Up to 50% of the parameters were practically identifiable within the tested range. Thus, our model ensemble is a good basis for future endeavours to provide a completely identifiable model.

When DBSs are measured with γH2AX-based technology it is important to include a term for not necessarily DSB-related background damage. Despite the precision of this readout its limitation is that phosphorylated γH2AX formation can occur at single-stranded DNA regions, which arise during replication or repair and, thus, does not solely correlate with DSB formation^21^,^39^,^40^,^41^. Not including this term would overestimate radiation included DSBs and especially TAFs. We accounted for this issue in the model by the parameter *B*. Estimation of this parameter suggested an average background of about one focus per cell.

We found that a nonlinear square-root relationship could better explain the observed dose-TAF relationship than the usually assumed linear dose-DNA damage relationship. Interestingly, this was also the fact for total γH2AX foci, despite several reports that propose a linear dose-response^1^,^2^,^10^,^22^,^23^. Our model discrimination results are in line with recent findings that shed doubt on the concept on a linear dose-foci relationship^42^. It has been observed that especially for high radiation dose DNA damage foci start to cluster and form ‘repair-centres’, where probably multiple DSBs are handled in parallel. These repair-centres are potentially also related to complex DSBs^42^. Thus, linear extrapolation might overestimate both DNA damage and corresponding cancer risk.

The concept of persistent DNA damage at telomeres (TAF) is mainly based on physical principles, specifically the chance of a high-energy ionizing particle or photon hitting the telomeric region of the DNA. The amount of DNA is the same in all human cells and, therefore, the chance of radiation-induced TAF is cell type independent, which has been supported experimentally^17^. Therefore, our model is likely to predict well the amount of persistent DNA damage in any surviving cells after radiotherapy. However, it should be noted that other model parameters, like repair rates, are probably cell type specific. Interestingly, our predicted additional radiation-induced TAF(*T*_AF+_) corrected for background damage (*B*) correlated better to measured excess relative risk (ERR) of secondary leukemia after fractionated radiotherapy than other much more sophisticated models (Fig. 6). However, it should be noted that our model was parameterized to data using dose rates of about 2.5 Gy per min. This is also in the range that is used for fractionated radiotherapy, opposed to brachytheraphy that uses much lower dose rates and accumulated doses. It is known that both low dose rates and low overall doses have substantially different effects on cells than high dose rates or overall doses^43^,^44^. Therefore, we did not try to predict ERR for brachytherapy. However, given a) the simplicity of our approach to predict ERR using TAF, b) the fact that our parameterization of IR-induced TAF is likely to be cell-type independent, and c) the good correlation of TAF with ERR, we suggest that simulated TAF may serve as an additional measure to predict leukemia/cancer risk after radiotherapy using high dose rates. This may improve predicting risk dose dependency of ionizing radiation especially for long-term therapies with accumulated doses larger than 1.6 Gy. However, it remains to be investigated experimentally, whether radiotherapy-induced persistent telomeric DNA damage is significant and correlates to secondary cancer risk.

## Materials and Methods

### Cell cultures

MRC5 normal human fibroblasts (at population doublings between 22 and 27) were cultured in Dulbecco’s modified Eagle’s medium (D-MEM) supplemented with 10% fetal bovine serum (FBS)(Gibco), 100x MEM non-essential amino acids solution (Gibco) and penicillin-streptomycin (Gibco). Cells were kept at 37 °C under 5% CO_2_ atmosphere and 95% humidity.

### Induction of DNA damage

DNA damage was induced by γ-irradiation: human primary fibroblast cells were exposed to ionizing radiation in a Biobeam GM 2000 (Gamma Medical Service) with^137^Cs as radioactive isotope and a dose rate of approximately 2.5 Gy/min.

### Immunofluorescent staining of γH2AX foci and image acquisition

Analysis of the DNA damage indicator γH2AX was determined by immuno-fluorescence at the single cell level. Cells grown on cover slips were washed in PBS and fixed in 4% paraformaldehyde (in 1xPBS, pH 7.4) for 15 min at room temperature. After three washing steps with PBS, cells were permeabilized using 0.1% Triton-X 100 (in 1xPBS, pH 7.4) for 15 min at room temperature and then incubated with the blocking reagent (5% Bovine serum albumin in 1xPBS, pH 7.4) for 45 min. The primary antibody anti-γH2AX (Ab26350, Abcam) was diluted to 1:1000 in 1% Bovine serum albumin, (in1xPBS pH 7.4) and added to the cells for 2 hours at room temperature. After the incubation, cells on cover slips were washed three times in PBS and the fluorescent-labelled secondary antibody (1:500) was added to the cells (IgG-Alexa488, Cell Sinaling #4408). The samples were stored in the dark at room temperature for 1 h. After washing, the DNA was stained with 49-6-diamidine-2-phenyl indole (DAPI, Invitrogen) diluted to 1 μg/ml in the same buffer for 5 min at room temperature. Cells were then washed in PBS and 10 μl of antifade medium (Vectashield) was dropped onto clean slides and the cover slips were transferred onto the slides and fixed with nail polish. Then MRC-5 cells were imaged using a confocal fluorescent laser scanning microscope (FluoView1000, Olympus) with a 60×oil objective. For the quantification procedure of γH2AX foci, please refer to the Supplementary Material (Image Quantification section and Figures S7 and S8).

### Model implementation, parameterization, and discrimination

Models were implemented as ordinary differential-algebraic equations using COPASI^45^. The free parameters were fitted to the data using COPASI’s Evolutionary Programming algorithm with population size of 10 times the number of parameters, and generation number of 10 times the population size. As objective function we employed the weighted sum of squared residuals:


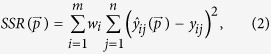


with *i* = 1,…, *m* as the number of experiments, and *j* = 1,…, *n* as the data points for experiment *i. w*_i_ represents the respective weight of experiment *i*, set to the inverse of the average of the respective time series. 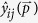 is the simulated value for data point number *j* within experiment *i* depending on the parameter vector 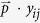 is the measured data point *j* within experiment *i*.

For model ranking (Table 1), we calculated the Akaike Information Criterion corrected for small sample sizes (*AICc*)^26^ for each candidate model:


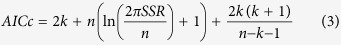


where *SSR* is the sum of squared residuals of the fit, *k* the number of parameters, and *n* the number of data points. The *AICc* is an information-theory based measure of parsimonious data representation that incorporates the goodness of the fit (*SSR*) as well as the complexity of the model (*k*), thereby giving an objective measure for model selection and discrimination.

In order to assess the evidence in favour of the best ranked model(s), provided by the data, we calculated Akaike weights (*AICw*)^26^.


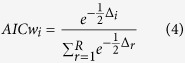


Where Δ_*i*_ *=* *AIC*_*i*_ − *AIC*_*min*,_ with *i* being the model index *i* = 1… *20* according to ranking and *AIC*_*min*_ the minimal *AICc*. The *AICw’s* can be considered as the weight of evidence in favour of a model given as a number between 0 and 1. Thus, the higher the weight, the higher the chance of a correct model selection. Models with *AICw* > *0.125* (cutoff) were considered as most probable candidates^26^.

### Crossvalidation

We assessed the predictive power of the models using a simple cross-validation strategy^46^,^47^; we divided the data into training and test data sets. The training data were used for model calibration while the test data were used for assessing the predictive power of the respective model. The telomere-associated DNA damage foci (TAF) data (Fig. 2E, S2) were always part of the training data, because it was the only information available for estimating TAF. For each cross-validation run, one of the remaining five data sets (Fig. 2A–D,F) was used for calculating the respective prediction error using the weighted sum of squared residuals between model simulation and test data. After five runs each data set was predicted once and the overall predictive power of the model was assessed by calculating the average prediction error (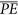):


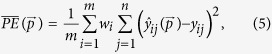


with *i* = 1,…, *m* as the number of the test experiment dedicated for prediction error calculation, and *j* = 1,…, *n* as the data points for the test experiment *i*. *w*_i_ represents the respective weight of experiment *i*, set to the inverse of the average of the respective time series. 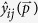 is the simulated value for data point number *j* within the test experiment *i* depending on the parameter vector 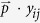 is the measured data point *j* within the test experiment *i*.

### Robustness analysis

We analysed the robustness of the optimal solution for the best approximating model Nr. 10 with respect to noise in the parameters including initial values. To this end, we performed a Monte-Carlo analysis by Latin hypercube sampling parameter values within ±20% of their respective fitted values for 1000 times using the Matlab statistics toolbox function, *lhsdesign*. Then, we simulated the model using the perturbed parameter sets (Fig. 5a), and calculated the relative standard deviation from the optimal solution (Fig. 5a- Inset) and repair time for 2.5, 10 and 20 Gy IR (Fig. 5b).

We quantified the relative standard deviation *σ*_sim_(*t*) of the simulated H2AX values from the optimal solution as


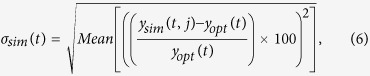


where *y*_*sim*_(*t, j*) and *y*_*opt*_(*t*) are model simulations and optimal solution as a function of time *t* and simulation number *j* (*j* = 1,…, *n*), respectively.

The repair time *τ* was defined as the time when the simulated yH2AX foci reached 150% of their respective final value. The latter was calculated as *B* + *T*_AF_ (*Gy*).

## Additional Information

**How to cite this article**: Rastgou Talemi, S. *et al.* Development of a robust DNA damage model including persistent telomere-associated damage with application to secondary cancer risk assessment. *Sci. Rep.* **5**, 13540; doi: 10.1038/srep13540 (2015).

## Supplementary Material

Supplementary Information

## Figures and Tables

**Table 1:** Model ranking.

Rank	ModelNr.	*P*(0)	2^nd^	*S,C*	*k*_2,4_	Lin/Sq	n	k	SSR	*AICc*	*AICw*	cutoff
1	10	20	2^nd^	*k*_**f,s**_	—	Sq	49	10	40.9	156.0	0.730	Yes
2	18	*P*_0_	2^nd^	*k*_**f,s**_	—	Sq	49	11	40.0	158.3	0.236	Yes
3	2	20	2^nd^	*k*_**f,s**_	*k*_2,4_	Sq	49	12	40.9	162.9	0.023	No
4	14	*P*_0_	2^nd^	*k*_**f,s**_	*k*_2,4_	Sq	49	13	39.2	164.5	0.010	No
5	8	—	—	—	—	Sq	49	7	80.8	180.3	0	No
6	6	—	2^nd^	—	—	Sq	49	8	77.3	181.0	0	No
7	12	20	—	*k*_**f,s**_	—	Sq	49	9	72.7	181.0	0	No
8	20	*P*_0_	—	*k*_**f,s**_	—	Sq	49	10	72.5	184.0	0	No
9	4	20	—	*k*_**f,s**_	*k*_2,4_	Sq	49	11	72.4	187.3	0	No
10	9	20	2^nd^	*k*_**f,s**_	—	Lin	49	10	83.0	190.7	0	No
11	17	*P*_0_	2^nd^	*k*_**f,s**_	—	Lin	49	11	83.0	194.0	0	No
12	13	*P*_0_	2^nd^	*k*_**f,s**_	*k*_2,4_	Lin	49	13	71.6	194.0	0	No
13	1	20	2^nd^	*k*_**f,s**_	*k*_2,4_	Lin	49	12	83.0	197.5	0	No
14	16	*P*_0_	—	*k*_**f,s**_	*k*_2,4_	Sq	49	12	91.7	202.4	0	No
15	5	—	2^nd^	—	—	Lin	49	8	169.6	219.5	0	No
16	7	—	—	—	—	Lin	49	7	285.0	242.1	0	No
17	11	20	—	*k*_**f,s**_	—	Lin	49	9	285.1	248.0	0	No
18	19	*P*_0_	—	*k*_**f,s**_	—	Lin	49	10	285.2	251.1	0	No
19	3	20	—	*k*_**f,s**_	*k*_2,4_	Lin	49	11	285.1	254.5	0	No
20	15	*P*_0_	—	*k*_**f,s**_	*k*_2,4_	Lin	49	12	285.1	258.0	0	No

*P*(0): initial condition for repair proteins, 2^nd^: with or without 2^nd^-order reaction, *S,C*: with or without protein-bound complexes, *k*_2,4_: with or without backward protein-binding reactions, Lin/Sq: Linear or square-root relationship between ionizing radiation (D) and initial DSBs/TAF, n: number of fitted data points, SSR: sum of squared residuals, *AICc*: Akaike Information Criterion adapted to small sample size, *AIC-w*: Akaike weights, Cutoff: indicates whether the model is within the cutoff region for acceptable models (Materials and Methods).

**Table 2:** Models average prediction error (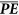).

Rank	Model Nr.	( 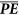 )
1	18	46.2
2	10	46.7
3	2	65.6
4	12	92.4
5	4	94.6
6	16	95.6
7	8	97.1
8	6	105.8
9	14	112.4
10	20	165.3
11	5	699.1
12	11	943.9
13	15	945.2
14	7	947.4
15	3	948.0
16	19	948.3
17	1	1680.6
18	9	1703.3
19	13	1708.8
20	17	1712.0

The average prediction error (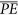) was calculated for all models using a simple cross cross-validation strategy (see Materials and Methods). The models are ranked based on 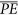 in an ascending manner.
