# Supplementary material for: Development of a robust DNA damage model including persistent telomere-associated damage with application to secondary cancer risk assessment
Source: Sci Rep. 2015 Sep 11;5:13540. doi: 10.1038/srep13540 (PMC4566481; doi:10.1038/srep13540)
Supplement: Supplementary Information [file srep13540-s1.pdf]

Supplementary Material

Development of a robust DNA damage model including persistent telomere-associated damage with application to secondary cancer risk assessment

Soheil Rastgou Talemi[1], Gabriel Kollarovic[1,2], Anastasiya Lapytsko[1], Jörg Schaber[1*]

[1] Institute for Experimental Internal Medicine, Medical Faculty, Otto von Guericke University, Magdeburg, Germany

[2] Cancer Research Institute, Slovak Academy of Sciences, Bratislava, Slovakia

[*] To whom correspondence should be addressed:

Jörg Schaber
Phone: +49 391 67 50227
Fax: +49 391 67 13312
Email: schaber@med.ovgu.de

Model Formulation

The models are implemented in systems of differential equations with events:

$$\frac{dS_i(t)}{dt} = -[P(t)]S_i(t)\left(\boldsymbol{k_1} + \left[\boldsymbol{k_{cross}}\left(S_i(t) + C_i(t)\right)\right]\right) + [\boldsymbol{k_2}S(t)]$$

$$\frac{dC_i(t)}{dt} = -[P(t)]C_i(t)\left(\boldsymbol{k_3} + \left[\boldsymbol{k_{cross}}\left(S_i(t) + C_i(t)\right)\right]\right) + [\boldsymbol{k_4}C(t)]$$

$$\frac{dS(t)}{dt} = [P(t)]S_i(t)\left(\boldsymbol{k_1} + \left[\boldsymbol{k_{cross}}\left(S_i(t) + C_i(t)\right)\right]\right) - \left([\boldsymbol{k_2}] + [\boldsymbol{k_f}]\right)S(t)$$

$$\frac{dC(t)}{dt} = [P(t)]C_i(t)\left(\boldsymbol{k_3} + \left[\boldsymbol{k_{cross}}\left(S_i(t) + C_i(t)\right)\right]\right) - ([\boldsymbol{k_4}] + [\boldsymbol{k_s}])C(t)$$

$$\begin{aligned}\frac{dP(t)}{dt} = & -[P(t)]S_i(t)\left(\boldsymbol{k_1} + \left[\boldsymbol{k_{cross}}\left(S_i(t) + C_i(t)\right)\right]\right) \\ & - [P(t)]C_i(t)\left(\boldsymbol{k_3} + \left[\boldsymbol{k_{cross}}\left(S_i(t) + C_i(t)\right)\right]\right) + \left([\boldsymbol{k_2}] + [\boldsymbol{k_f}]\right)S(t) \\ & + ([\boldsymbol{k_4}] + [\boldsymbol{k_s}])C(t)\end{aligned}$$

Event: $S_i = \begin{cases} 0 & ,t = 0 \\ (1 - \boldsymbol{c})\boldsymbol{k_{DSB}}\langle D, \sqrt{D}\rangle & ,t > 0 \end{cases}$

Event: $C_i = \begin{cases} 0 & ,t = 0 \\ \boldsymbol{c}\boldsymbol{k_{DSB}}\langle D, \sqrt{D}\rangle & ,t > 0 \end{cases}$

Event: $T_{AF}(t) = \begin{cases} \boldsymbol{T_0} & ,t = 0 \\ \boldsymbol{T_0} + \boldsymbol{k_{TAF}}\langle D, \sqrt{D}\rangle & ,t > 0 \end{cases}$

$P(0) = \langle 20, \boldsymbol{P_0}\rangle$

$F_t = \boldsymbol{B} + S_i(t) + C_i(t) + [S(t) + C(t)]$,

where *c* determines the percentage of initial complex DSBs, k_{DSB} and k_{TAF} determine the number of DSBs and TAF per absorbed irradiation dose *D* [Gy], respectively, square brackets [] indicate optional parameters/variables, and <,> indicate alternatives. The characteristics of the candidate models are listed in Table 1, where column names indicate alternative parameters and components. Bold model components indicate the corresponding free parameters that were either fitted to data or set to zero, depending on the model candidate. All other components were either set or calculated. At $t > 0$ T_{AF}, S_{i} and C_{i} were discretely set from their initial values to new values using respective events. The state variables have the unit ‘average number per cell’.

Supplementary Tables and Figures

Table S1: Estimated parameters and their estimated asymptotic standard deviation for the best approximating model Nr. 10.

Parameter	Value±SE	Unit	Description	Method
k_1	0.00535262±0.00175772	$\frac{1}{hour \cdot \#}$	First order kinetics, *S-P* association rate constant.	Estimated
k_2	0	$\frac{1}{hour \cdot \#}$	First order kinetics, S_i dissociation rate constant.	Not present in model Nr. 10
k_3	0.000467362±0.0085878	$\frac{1}{hour \cdot \#}$	First order kinetics, *C-P* association rate constant.	Estimated
k_4	0	$\frac{1}{hour \cdot \#}$	First order kinetics, C_i dissociation rate constant.	Not present in model Nr. 10
k_{cross}	0.000151845±0.0000274726	$\frac{1}{hour \cdot \#^2}$	Second-order, *S,C-P* association rate constant.	Estimated
k_s	0.00511497±0.00246494	$\frac{1}{hour}$	S_i repair rate constant.	Estimated
k_f	2000± 35166	$\frac{1}{hour}$	C_i repair rate constant.	Estimated
k_{DSB}	167.817± 21.4137	$\frac{\#}{Gy}$	Conversion constant of radiation dose to the number of radiation-induced DSBs.	Estimated
k_{TAF}	0.790502± 0.12566	$\frac{\#}{Gy}$	Conversion constant of radiation dose to the number T_{AF}.	Estimated
T_0	0.889883± 0.380939	#	Initial T_{AF} in non-radiated cells.	Estimated
B	0.989191± 0.535249	#	Average basal background DSB per cell in non-radiated cells.	Estimated
c	0.0225659±0.00370192	–	Initial C/(S+C), i.e. after radiation	Estimated

#: average number/amount of respective variable

Table S2: Initial values and auxiliary parameters.

Variables/Parameters	Value	Unit	Description	Method
$S(0)$	0	#	Simple DNA damage-repair protein complex	Set
$S_i(0)$	0	#	Radiation induced simple DNA damage foci	Set
$C(0)$	0	#	Complex DNA damage-repair protein complex	Set
$C_i(0)$	0	#	Radiation induced complex DNA damage foci	Set
P_0	20	#	Initial repair protein number	fixed
F_t	$B + F_i + [S(t) + C(t)]$	#	Total DNA damage foci	Calculation

#: average number/amount of respective variable

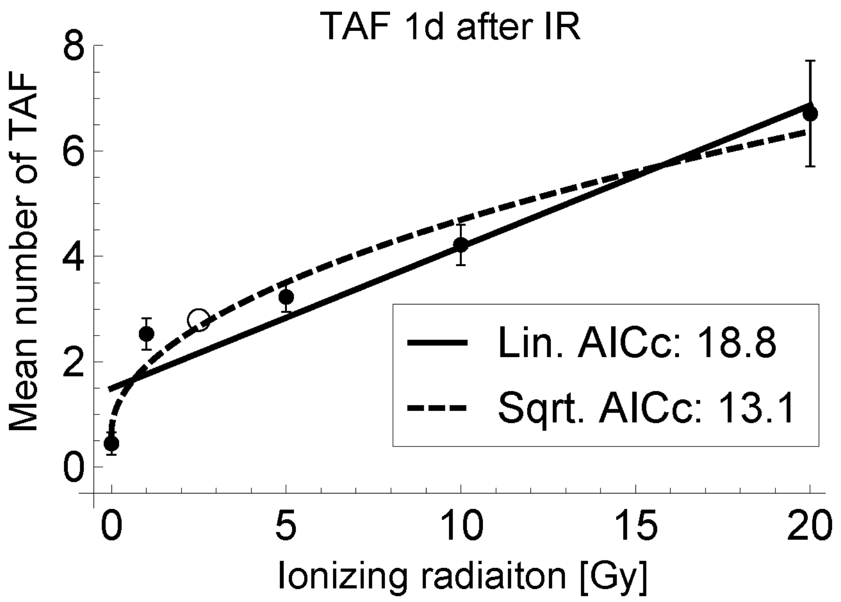

Figure S1: Measured mean±SEM number of telomere-associated foci (TAF) with fitted linear (solid line) and square-root (dashed line) function. Model selection using AICc favors square-root relationship. AICc: Akaike information criterion corrected for small sample size. Data from [1].

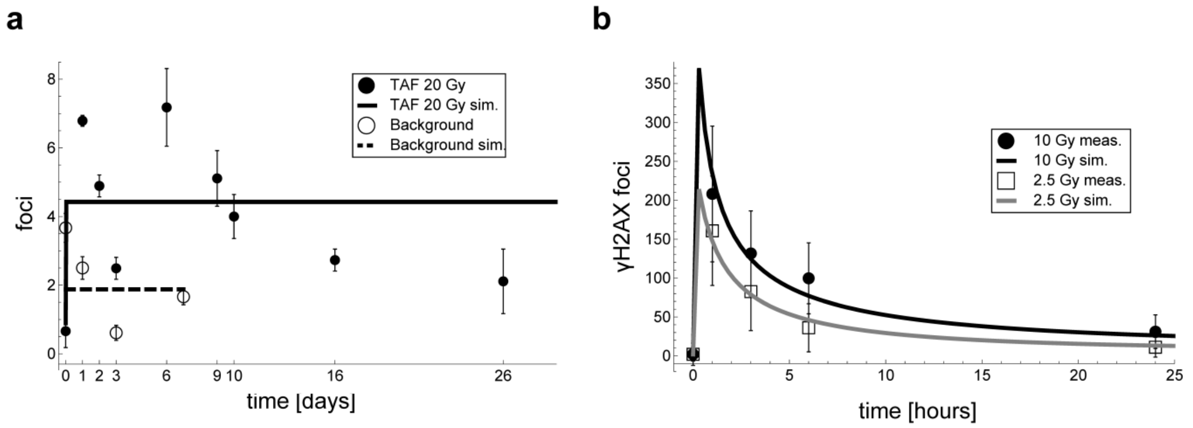

Figure S2: Additional model fits and simulations. **a**) Measured mean±SEM (symbols) and fitted (lines) values of background γH2AX foci per cell (open circles and dotted line) and telomere-associated foci (TAF) per cell after 20 Gy radiation (dots and full line). **b**) Measured mean±SEM (symbols) and fitted (lines) time series of γH2AX foci per cell after 10 (dots and black line) and 2.5 (open rectangles and gray line) Gy.

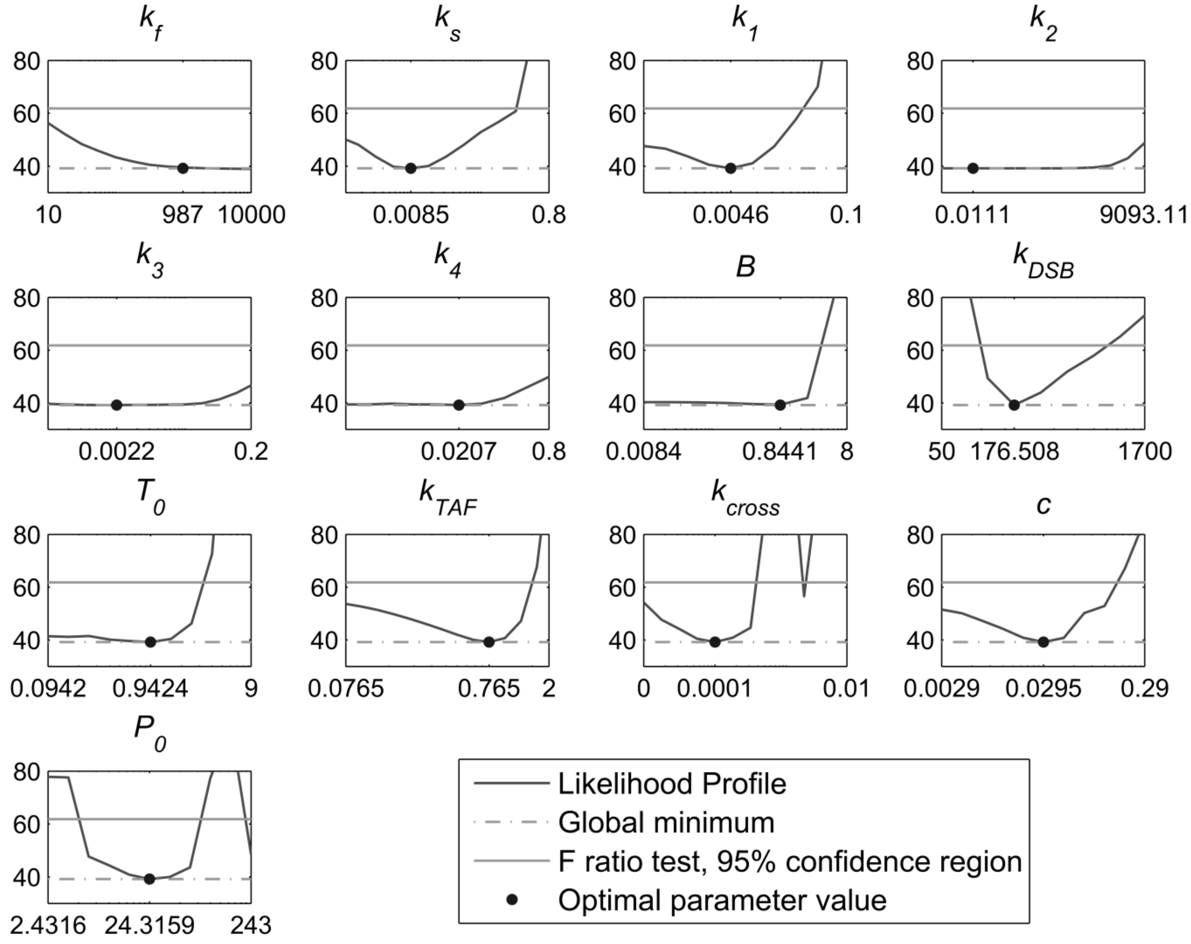

Figure S3: Profile likelihood-based parameter identifiability analysis for model Nr. 14. The SSR after parameter estimation is plotted versus scanned parameter values. 95% confidence region is calculated by F-ratio test (grey solid line). The minimum objective value reached is shown at bottom (grey dashed line) and the corresponding estimated parameter value is shown by a bold dot. Parameter identifiability analysis results showed no structural non-

identifiability, absence of a flat SSR profile, in model Nr. 14. It also indicates that 4 parameters out of 13 are practically identifiable.

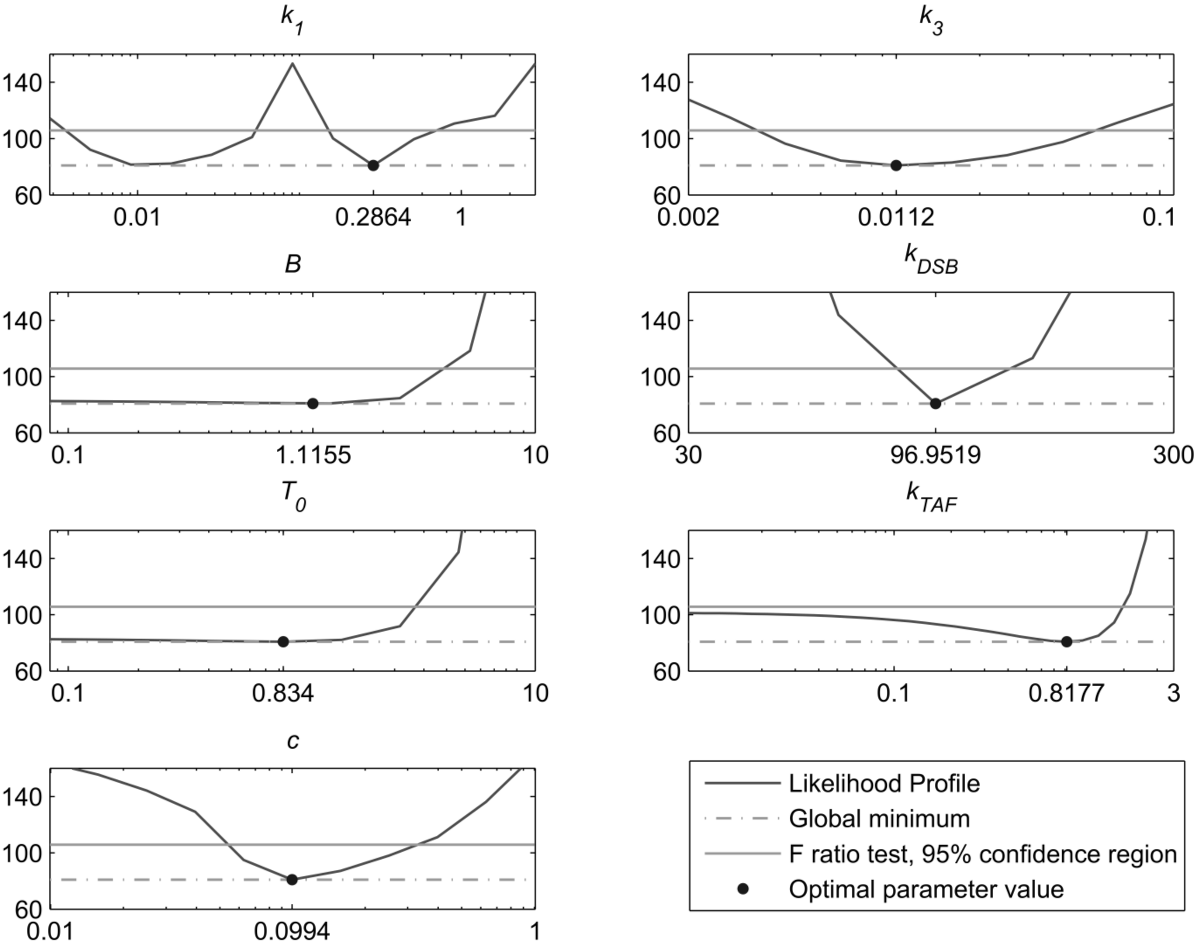

Figure S4: Profile likelihood-based parameter identifiability analysis for models Nr. 8. The SSR after parameter estimation is plotted versus scanned parameter values. 95% confidence region is calculated by F-ratio test (grey solid line). The minimum objective value reached is shown at bottom (grey dashed line) and the corresponding estimated parameter value is shown by a bold dot. Parameter identifiability analysis results showed no structural non-identifiability, absence of a flat SSR profile, in model Nr. 8. This indicates that 5 parameters out of 7 are practically identifiable.

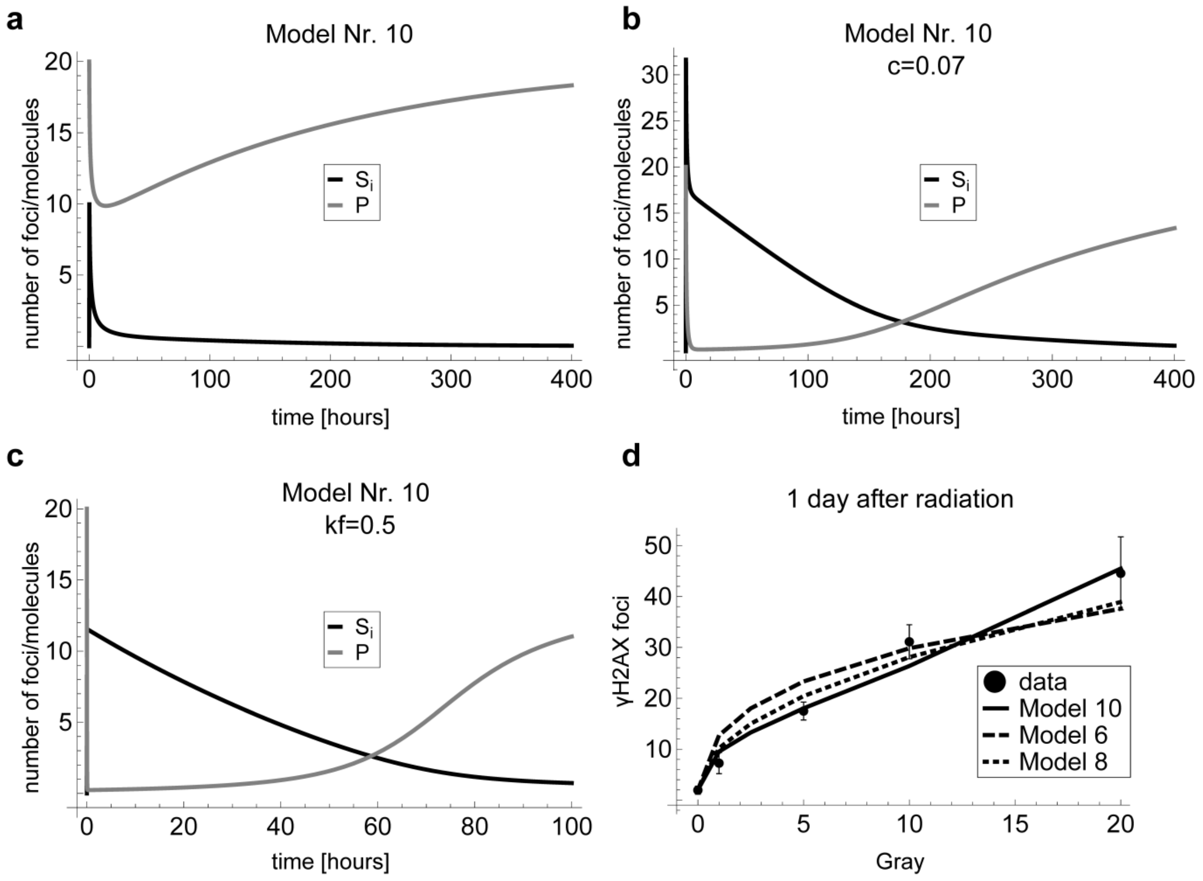

Figure S5: Model analyses for the best approximating model Nr. 10. **a-c**: Simulations of simple DSBs (S_i) and free repair proteins (P) after 10 Gy for different parameter settings. **d**) simulations of dose [Gy]-response (mean±SEM γH2AX foci per cell) relationship 24h after radiation for different models.

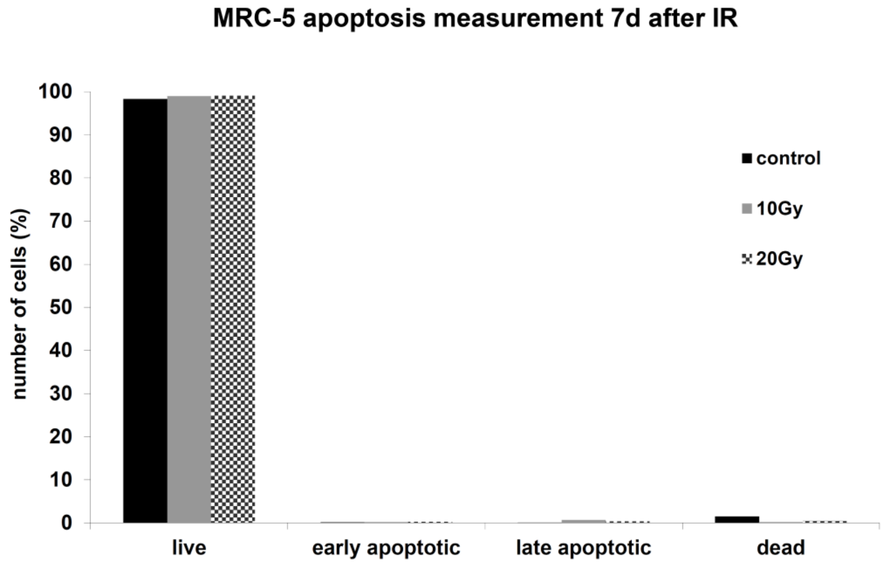

Figure S6: MRC5 apoptosis is negligible after irradiation. MRC5 cells were exposed to no irradiation (control), or ionizing radiation of 10Gy and 20Gy and incubated seven days. Afterwards the cells were harvested and stained with annexin V-FITC and propidium iodide (PI), and they were subjected to flow cytometry. Percentage of live cells (negative for both annexin V and PI), early apoptotic cells (positive for annexin V and negative for PI), late apoptotic/necrotic cells (positive for both annexin V and PI) and dead cells (negative for

annexin V and positive for PI) were determined. Results are expressed as a mean of one experiment.

Annexin-V/ Propidium Iodide flow cytometry analysis

Apoptosis was determined by using Annexin V-FLUOS Staining Kit (Roche) according to the manufacturer's instructions. Briefly, MRC-5 primary human fibroblasts both non-radiated and irradiated either with 10 or 20 Gy were washed with 1x PBS, harvested by trypsinization and centrifuged at 200 x *g*. The cell pellet ($1x10^6$) was resuspended in 100 µl of the Annexin-V-FLUOS labeling solution (2 µl Annexin-V-Fluos reagent and 2 µl Propidium iodide solution in 100 µl of incubation buffer) and incubated for 15 min at room temperature. For FACS analysis, 500 µl of the incubation buffer was added into the labeled cells. Samples were analyzed by using the CyFlow space (Partec). Positive and negative controls (incubation buffer only, Propidium iodide (PI) only, Annexin V-Fluos only) were used to set up appropriate conditions.

Image Quantification

Quantification of γH2AX foci time series from published papers

We quantified the experimental results already published by Hewitt et al. [1] abd Fumagalli et al. [2] using the free software "Plot Digitizer" under the terms of the GNU General Public License.

Data scaling

The absolute number of quantified foci is hardly comparable among experiments from different laboratories, because of differences in the technical equipment, staining protocols and actual foci counting procedures. To make our data comparable to the data from Hewitt *et al.* (2012) we assumed that the total number of foci 24h after 10Gy radiation was the same in both experiments (Figure 2D) and scaled our data accordingly, excluding the initial time points, which were left unchanged. For scaling our 2.5 Gy time series we used the same approach, using a linear interpolation between measured γH2AX foci 24h after1 Gy and 5 Gy irradiation (Figure 2D, closed square).

Extended consensus γH2AX foci time series for 20 Gy

The γH2AX foci time series for 20 Gy was compiled using MRC5 data from Hewitt *et al.* (2012) and BJ data from Fumagalli *et al.* (2012). Fumagalli *et al.* (2012) focussed on long time series including 30, 60 and 120 days after IR, whereas Hewitt *et al.* (2012) mainly focused on earlier time points, i.e. until 26 days after IR. Fumagalli *et al.* (2012) show that the absolute amount of γH2AX foci after 10 days is almost indistinguishable between MRC5 and BJ cells (Supplementary Figure S2 therein). Therefore, we reasoned that especially the long-term data from Fumagalli's BJ cells can used for MRC5 cells as well. Moreover, there was no remarkable difference in absolute numbers between Hewitt's MRC5 data and Fumagalli's BJ data at later time points: for 10 days after 20 Gy both measured approximately 9 foci per nucleus on average. Therefore, we combined the two time series taking the average of both in case of overlapping measurements. Table S3 shows the combined time series with indicated data sources.

Table S3: Data of the *extended consensus γH2AX foci time series for 20 Gy*

Time [days]	Total	SEM
**0**	2.11	0.97
**1**	44.44	7.4
**2**	25.80	4.66
**3**	17.92	1.265
**6**	18.44	0.96
**9**	12.51	0.81
**10**	9.35	1.125
**16**	5.63	0.64
**26**	3.08	0.48
**30**	3.13	0.73
**60**	2.78	0.4
**120**	3.49	0.6

Data source
Average
Hewitt
Fumagalli

Quantification of γH2AX foci time series from fluorescent microscopy

The γH2AX foci quantification was performed automatically using custom written algorithm implemented in MatlabR2008b (see http://www.mathworks.com). In addition, we utilized Image Processing Toolbox of Matlab and ImageJ 1.45s as plug-in. ImageJ is a freely available image processing program that we obtained from http://imagej.nih.gov/ij/. For bi-directional communication and data exchange between Matlab and ImageJ we used a Java package MIJ obtained from the web page http://bigwww.epfl.ch/sage/soft/mij/.

The algorithm used for foci quantification includes following steps:

1. Convert an RGB image with both nuclei and foci to double precision and split into two channels: blue channel for getting nuclei image, green channel for getting foci image.
2. Create a nuclei mask applying following procedures to the nuclei image:
   - 2.1. correct possible non-uniform illumination using top-hat filtering (*imtophat* with structuring element *'disk'* of radius 3 pixels in Matlab).
   - 2.2. threshold using Li method (ij.IJ.run('Threshold...','Li') in ImageJ).
   - 2.3. exclude nuclei that touch an image border (*imclearborder* in Matlab).
   - 2.4. reduce a possible noise applying the median filter (*medfilt2* with default 3-by-3 neighborhood in Matlab).
   - 2.5. fill holes in nuclei if any exist (*imfill* in Matlab).
   - 2.6. erode and reconstruct using a structuring element *'disk'* with radius 10 pixels (*imerode* and *imreconstruct* in Matlab). This operation suppresses image elements that fit into the disk and preserves the rest image elements unchanged.
   - 2.7. restore the mask elements that have "c-shape" and that do not cover nuclei completely. For this we apply following operations:
     - 2.7.1. dilate 4 times (*bwmorph* with option *'dilate'* in Matlab).
     - 2.7.2. fill holes in the nuclei (*imfill* in Matlab).
     - 2.7.3. erode 4 times (*bwmorph* with option *'erode'* in Matlab).

2.8. apply watershed transformation to split touching nuclei (ij.IJ.run('Watershed') in ImageJ).

3. Label regions of the nuclei mask that belong to each recognized nucleus (*bwlabel* in Matlab).
4. Apply the nuclei mask to the foci image.
5. Crop the region of the foci image that belongs to a nucleus (*imcrop* in Matlab) and apply following transformations to perform foci counting:

7.1. apply top-hat filtering to remove background noise (*imtophat* with structuring element *'disk'* of radius 3 pixels).

7.2. calculate threshold T_{ot} using Otsu's method (*graythresh* in Matlab).

7.3. perform H-maxima transformation that suppresses maxima of the image with height less than T_{ot}. Then compute regional maxima of the H-maxima transformation. Both operations are combined in the function *imextendedmax* with parameter T_{ot} in Matlab.

7.4. find and count regions of regional maxima that have intensity values greater than estimated threshold value $T_e = 0.1$.

7.5. designate the number of these regions as the number of detected foci per nucleus.

In this study, we used average γH2AX foci count per cell nucleus to model the DNA damage response over time for different DNA damage levels. For counting we utilized images of immunofluorescent stained MRC5 cells (see Methods section). Images of cells were obtained at time points 1, 3, 6, 24, 48, 72, 168 hours after 2.5 Gy and 10 Gy irradiation within three repetitions. For each time point we measured the foci number in about 100 cell nuclei.

In Figure S7 and Figure S8 we visualized the result of foci count in nuclei cropped from representative images. Images were selected from three experimental repetitions. Foci, which were recognized and counted by the algorithm, are marked by red frames.

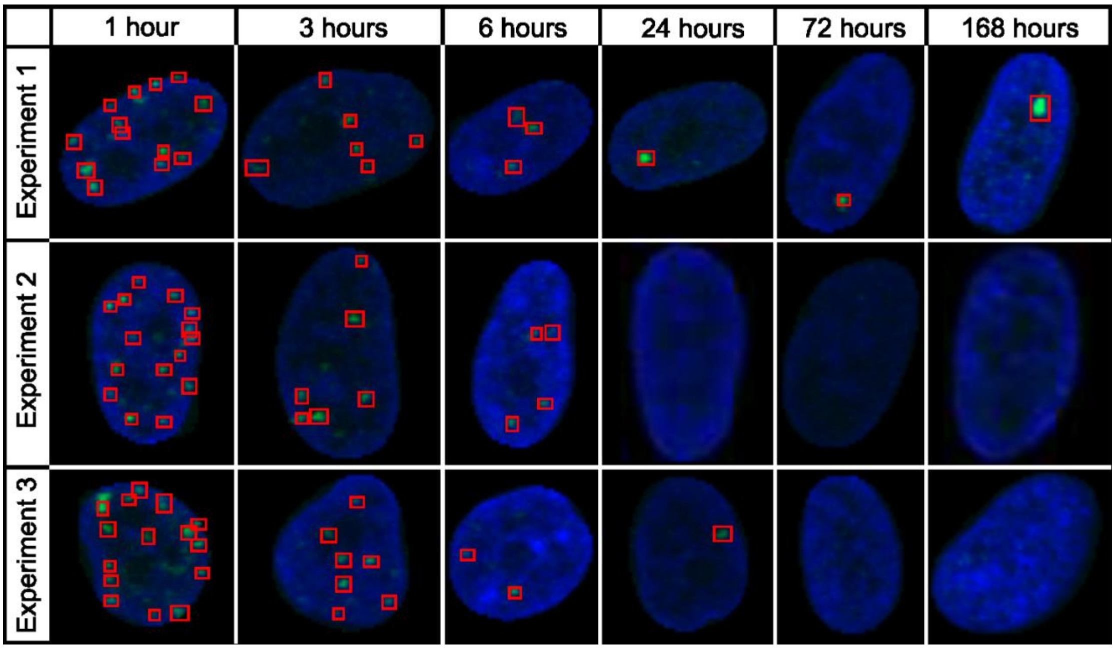

Figure S7: The visualization of foci count in nuclei of immunofluorescent stained MRC5 cells after 1, 3, 6, 24, 48, 72, 168 hours post 2.5 Gy irradiation. Images of nuclei were picked

from three experiment repetitions. Foci, which were recognized and counted by the algorithm, are marked by red frames.

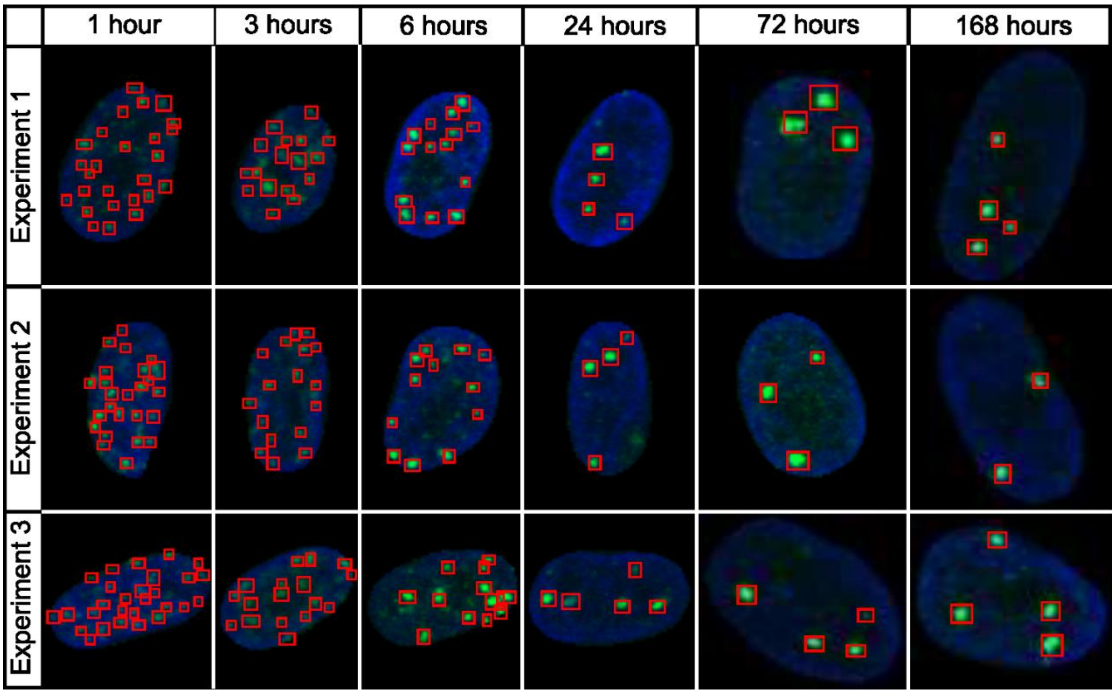

Figure S8: The visualization of foci count in nuclei of immunofluorescent stained MRC5 cells after 1, 3, 6, 24, 48, 72, 168 hours post 10 Gy irradiation. Images of nuclei were picked from three experiment repetitions. Foci, which were recognized and counted by the algorithm, are marked by red frames.

References

1. Hewitt G, Jurk D, Marques FD, Correia-Melo C, Hardy T, et al. (2012) Telomeres are favoured targets of a persistent DNA damage response in ageing and stress-induced senescence. Nat Commun 3: 708.
2. Fumagalli M, Rossiello F, Clerici M, Barozzi S, Cittaro D, et al. (2012) Telomeric DNA damage is irreparable and causes persistent DNA-damage-response activation. Nat Cell Biol 14: 355-365.
